# Accessibility Considerations in the National Children's Study

**DOI:** 10.3389/fped.2021.624175

**Published:** 2021-04-14

**Authors:** Mark Harniss, Susan Magasi, Dianne Sabat

**Affiliations:** ^1^Department of Rehabilitation Medicine, University of Washington, Seattle, WA, United States; ^2^Departments of Occupational Therapy and Disability and Human Development, University of Illinois at Chicago, Chicago, IL, United States; ^3^Mukilteo School District, Mukilteo, WA, United States

**Keywords:** accessibility, disability, outcomes measurement, measurement, test development

## Abstract

In the National Children's Study (NCS), assessments were proposed and developed that used a wide range of modes of administration (e.g., direct in-person interviews, telephone interviews, computer assisted interviews, self-administered questionnaires, real time and recall observations, and physical examinations). These modes of administration may pose accessibility challenges for some people with disabilities. Accessibility of measurement is important to consider because systematic exclusion of people with disabilities from research can lead to measurement bias and systematic error in derived scores. We describe our approach to analyzing the accessibility of measures in the NCS and describe the work of the Accessibility Domain Team. Finally, we describe a decision process for creating and using accessible health research measures.

## Introduction

Findings from the 2018-2019 National Survey of Children's Health indicate that 18.9% of children in the United States under the age of 18 years (or ~13.9 million children) have special health care needs (1). The more conservative American Community Survey indicates that 4.3% of the civilian non-institutionalized population of children aged under 18 have a disability (2). The Survey of Income and Program Participation shows that disability increases as children age. Disability is reported in 0.7% of children under the age of four; 5.3 % in children ages 5-15 and 5.9% in children ages 16-20. For children ages 5-15, disabilities cover a wide gamut and include cognitive disabilities (4.1%), difficulty seeing (0.8%), difficulty hearing (0.6%), difficulty with speech (2.0%), difficulty with running or walking (.6 %), and difficulty with dressing or bathing (1.0%) (2, 3). There is also increasing recognition of the prevalence of mental health disabilities among children and adolescents with approximately one in every four to five youth in the U.S. meeting the criteria for a mental disorder with severe impairment across their lifetime (4).

These estimates may be conservative. For example, the U.S. Department of Education reports 13.7% of children ages 3-21 being served in the public school system have varying disabilities, with learning disabilities being the most prevalent at 4.6% (5). Furthermore, a study by researchers at the Centers for Disease Control (CDC) and Prevention and the DC and Health Resources and Services Administration using data from the 1997–2008 National Health Interview Surveys suggests that between 1997 and 2008, the prevalence of any developmental disability was about 15% in children ages 3-17 (6). They also found that the prevalence of developmental disabilities has increased 17.1% over that time frame and that some disabilities had significant increases (e.g., autism 289.5%; ADHD 33%). According to the CDC's Autism and Developmental Disabilities Monitoring Network, autism is diagnosed in about 14.6% of children (7).

People with disabilities, including children with disabilities and special healthcare needs, are frequently excluded from clinical, epidemiological, and longitudinal research (8–11). When children with disabilities and special healthcare needs are excluded from research, they become missing data which compromises the ability of health systems to develop, implement and evaluate person-centered preventive, primary and long-term care services that address their health needs. The lack of accurate data on health and healthcare disparities among children with disabilities and special healthcare needs precludes the development of health policies and public health practices to address equity at individual, community, and population levels for people with disabilities. Furthermore, differences across population-based surveys in how disability and special health care needs are defined and operationalized lead to markedly different prevalence estimates, which can in turn influence decision and policy making (12). One reason for this exclusion is the lack of reliable, valid and responsive measures that are accessible to people with disabilities, in general, and children, in particular (9, 13–15). There is a lineage of population-based surveys that seek to provide information on the physical and emotional health of children in the United States, including for example the National Survey of Children with Special Health Care Needs (16) which was later consolidated with the National Survey of Children's Health (17). These cross-sectional telephone and web-based surveys provide a snapshot of children's health using a variety of self-report outcome and performance measures.

By comparison, the National Children's Study was designed as a prospective “longitudinal study of environmental influences on children's health and development” (18). The National Children's Study sought to extend beyond self-report of physical and emotional health to include capture of primary data when feasible. Therefore, rather than *asking* about disability and special health care needs, the National Children's Study was challenged with how to *include and ensure fairness in testing* for children and parents with disabilities and special health care needs. Both self-report and performance based measures have the potential to expand current understanding of children's health and development, and work toward achieving public health priorities for children with special health care needs and disabilities, like those outlined in the Healthy People initiatives since 2000 (19). It is critical to ensure that measurement instruments are accessible and usable for diverse respondents to ensure that they capture the health experiences and functional capacity of children with disabilities and special healthcare needs.

Population-based epidemiological and longitudinal studies of child development, such as the National Children's Study, required the identification, selection or development of performance-based measurement instruments that were reliable, valid, sensitive, and specific to children from diverse backgrounds across the age range. Furthermore, to ensure that all children, including those with disabilities, were able to demonstrate their standing on the constructs measured it was important that measurement instruments be accessible and universally designed for as broad a spectrum of functional abilities as possible (20). Indeed accessibility and universal design are closely related to the concept of fairness in testing, which the Standards for Educational and Psychological Testing (20) identify as a fundamental validity issue in all phases of test development, administration and use. Accessible measurement instruments should minimize construct-irrelevant components so that test takers are not “scored down” due to peripheral task demands like difficulties perceiving test stimuli or entering response options. For example, most computer-administered neurocognitive assessments require test takers to manually enter their responses via mouse click, touch screen or keyboard. Such a test may selectively disadvantage users with decreased motor control in their arms—even when motor control is not an element of the construct.

The leadership of the National Children's Study demonstrated a commitment to ensuring that all measurement instruments included in their testing batteries strove for the highest possible degree of accessibility. Specifically, within the Health Measurements Network, an Accessibility Working Group was created to collaborate with content and measurement experts to evaluate and improve the accessibility of all proposed measurement instruments (18). Harniss and Magasi have experience working in both the Patient Reported Outcomes Measurement Information System (PROMIS®) and NIH Toolbox® for Assessment of Neurological and Behavioral Function (NIH Toolbox, NIHTB) initiatives to improve measurement accessibility (9, 21–24). In this paper, we describe the approach and processes we used in the National Children Study's Accessibility Working Group to promote accessible measurement for children with disabilities and special healthcare needs. We include recommendations for both developers of measurement instruments and users of these instruments to promote accessible and inclusive practices.

## Analyzing the Accessibility of Measures

Measures can be analyzed by considering task demands within the context of the measure's purpose and the primary construct of interest. We categorized a measure's accessibility in five categories.

1. **Accessible as Developed**. Includes measures that are accessible as developed for a specific individual or group.
2. **Accessible with Accommodation as Standard Administration**. Includes measures that are inaccessible to some users in their current form but can be made accessible with reasonable accommodations as a *standard* administration. A standard administration is one in which the data are comparable and can be combined in analysis. For example, the NIHTB Odor Identification Test presents stimuli using “scratch and sniff” cards. If an individual cannot scratch and sniff the cards independently, the administrator can scratch the card. This would be considered a standard administration because it does not alter the construct being measured.
3. **Accessible with Accommodation as Non-standard Administration**. Includes measures that are inaccessible to some users in their current form but can be made accessible with reasonable accommodations in a *non-standard* administration. Non-standard administration refers to changes in administration protocols that alter the underlying construct. For example, emotional health PRO items assessing depression or pain can be made accessible to individuals who are blind, have low vision, or reading disabilities by having the test administrator read the items out loud. This would not be a standard administration because participants might adjust their responses in a socially desirable manner. There are two benefits of deciding to use a non-standard administration before beginning a research study. First, non-standard administrations may not be included in the larger analysis, but they can be compared in sub-analyses. Second, considering the possibilities for non-standard administration a *priori* reduces the chance that participants will attempt to complete a measure in a way that was not intended by the test developers and that invalidates the results. For example, the NIHTB Pattern Comparison test measures processing speed and mental efficiency. The test is timed, and speed is a component of the score. The test requires quickly hitting a left or right arrow key. An individual with quadriplegia may be able to complete this measure by compensating for lack of isolated finger movement with gross motor movements. Functionally, she or he may be able to finish the measure but slowly and with more errors. The results may accurately reflect motor function and not processing speed. This measure can be completed, but it is not accessible because the scores are not valid for the intended purpose. An alternate measure would be needed to include participants with fine motor limitations.
4. **Not Accessible because of Secondary Task Demands**. This category includes measures that are inaccessible to some individuals because they have secondary task demands that assess a construct not related to a functional deficit. That is, they have construct-irrelevant variance. For example, if endurance is measured by a 2-min walk test, wheelchair users cannot demonstrate their endurance. An alternate measure, like the 6-Min Push Test, would be needed to for participants who use wheelchairs (25).
5. **Not Appropriate because It Directly Measures a Functional Deficit**. Includes measures that are inaccessible to some individuals because they assess a construct directly related to a functional deficit (e.g., an ambulation test for people who use wheelchairs, a vision test for people who are blind). These measures are valid for their intended use but are not appropriate for specific populations.

**Figure 1:**
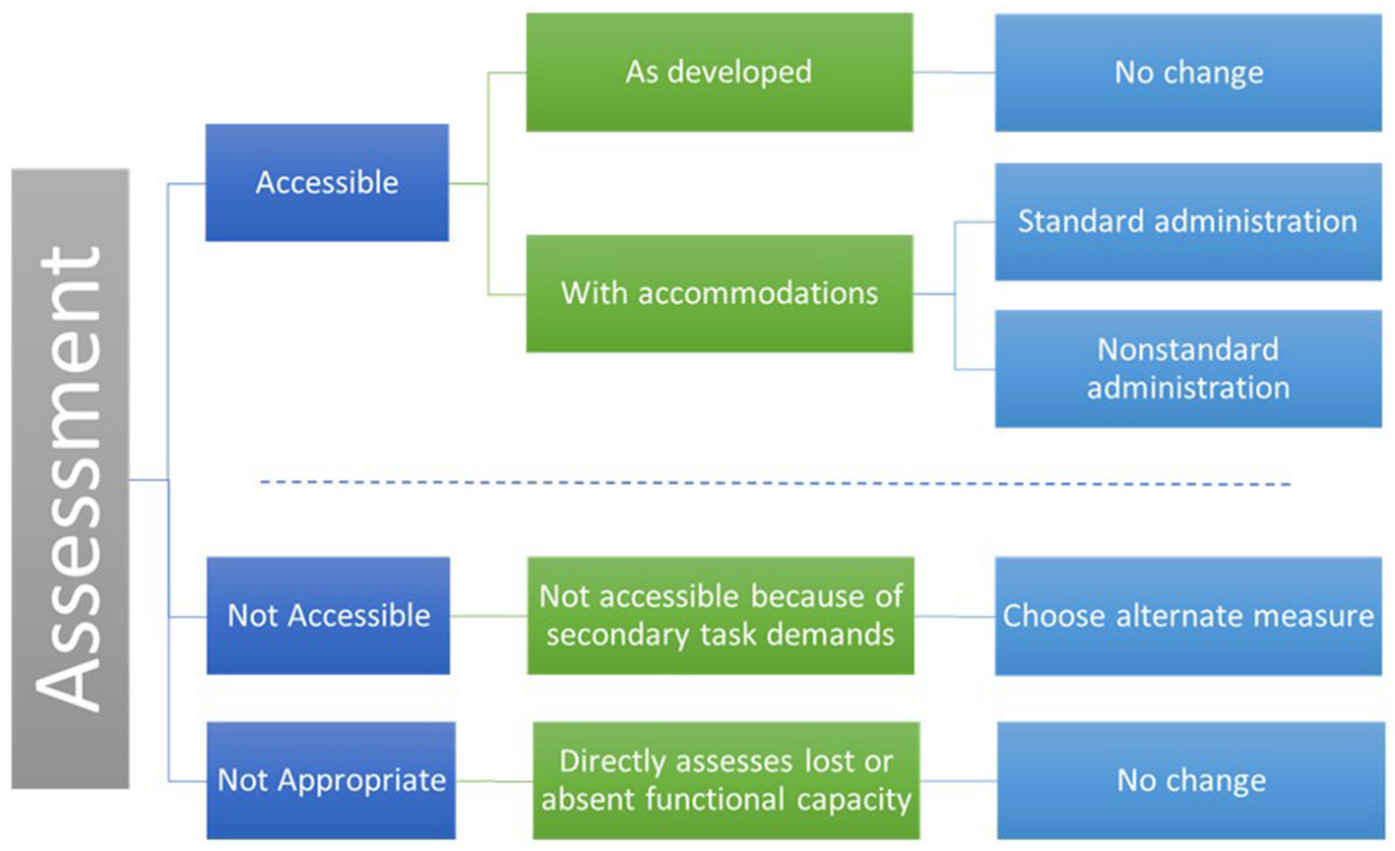
Accessibility categorization of measures.

Figure 1 depicts the five ways measure can be categorized.

## NCS Accessibility Domain Team Goals

The original scope of the accessibility work group included the following:

1. Knowledge translation to increase accessibility of measures in development.
2. Accessibility review of existing measures.
3. Research to evaluate effectiveness.

After the NCS was closed by NIH, our scope of work was revised to include the following:

- Provide feedback from our reviews of the new measurement proposals and continue working with domain teams who were approved to move forward with development.
- Review the iPad versions of the NIHTB, including the development of a reasonable accommodations guideline for iPad administration.
- Complete a conceptual white paper for publication on the topic of accessible measurement.

### Knowledge Translation

In the early stages of the project, members of the Accessibility Work Group presented an hour-long training to each domain team addressing the rationale for considering accessibility in the NCS. Trainings defined accessibility within the context of measurement development and provided a process for evaluating accessibility of newly developed measures. This content was integrated into a Webinar “MeasureABLE: Enhancing Accessible Outcomes Measurement for People with Disabilities.” These trainings were used to increase awareness of the importance of accessibility and inclusion of people with disabilities in research.

The Work Group also recruited an accessibility champion from each domain team to help promote issues of access and inclusion in the instrument development and selection process. We met with the leaders of the other special working groups (i.e., multicultural team, translations team) to develop a harmonized review process.

### Accessibility Feedback to Domain Teams

#### Review of New Measure Proposals

The Accessibility Work Group reviewed the proposals for all measures that had been submitted to the NCS leadership for development or adaptation and evaluated them in terms of accessibility. We first reviewed short proposals that provided an overview of the development process. Based on our understanding of the measure from the proposal, we generated accessibility concerns and questions. Domain teams responded to our questions with clarifying information. We then generated accessibility concerns and solutions based on the functional demands of the measure.

The accessibility feedback provided to the development teams was intended to guide future development of their measures in two ways. First, some of the feedback might have resulted in changes to the measures themselves to reduce accessibility barriers and increase the likelihood that people with various functional deficits can be measured accurately. Second, feedback might have resulted in changes to the approved accommodations that were allowed during implementation of the measures. Standardizing acceptable accommodations increases consistent application over a range of test administrators.

#### Accessibility Review of the iPad Version of The NIH Toolbox

Our team reviewed the original version of the NIHTB when it was implemented using a dual screen administration with a laptop and external monitor. The original Accommodations Manual can be found here: http://www.healthmeasures.net/2-uncategorised/209-nih-toolbox-technical-manuals-for-ac. For the NCS, we compared our accessibility recommendations for the computer-based NIHTB to a new system implemented on iPad. Since we only had access to the cognition and emotion measures, our review was limited to those domains (the motor and sensory domain batteries were scheduled for release after the termination of the NCS contract in the fall of 2015). The review was based on expert opinion following a hands-on evaluation of the measures on the iPad platform. All findings need confirmation through accessibility testing with a range of users with disabilities.

We presented our findings across four categories, (1) functional requirements of the measure, (2) accessibility issues, (3) recommendations, and (4) groups for whom accessibility is likely not possible. Based on this review, we also adapted and published an iPad version of the NIHTB Accommodations Manual.

#### White Papers

We wrote two white papers related to our work. The first paper addressed concepts of accessibility as they relate to measurement, ways in which accessibility decisions both increase the validity of measurement and challenge the standardization of research designs, and strategies for analyzing the accessibility of measures. In addition to the concepts found in this paper, issues of accessibility and inclusion of people with disabilities in research emerged as an important issue in the course of our work. We wrote a second white paper on the topic of accessible research procedures that has been published in the American Journal of Public Health (9). This paper addresses accessibility decisions throughout the research process including accessible recruitment, consent, facilities, measurement, and interventions.

## An Accessibility Decision Process for Test Developers and Researchers

A prerequisite for the creation of accessible health measures and measurement systems is the recognition that accessibility and fairness in testing are important validity considerations at all phases of test development, administration, and interpretation. In this section, we describe a decision process for test developers and researchers as they create and select measures.

**Figure 2:**
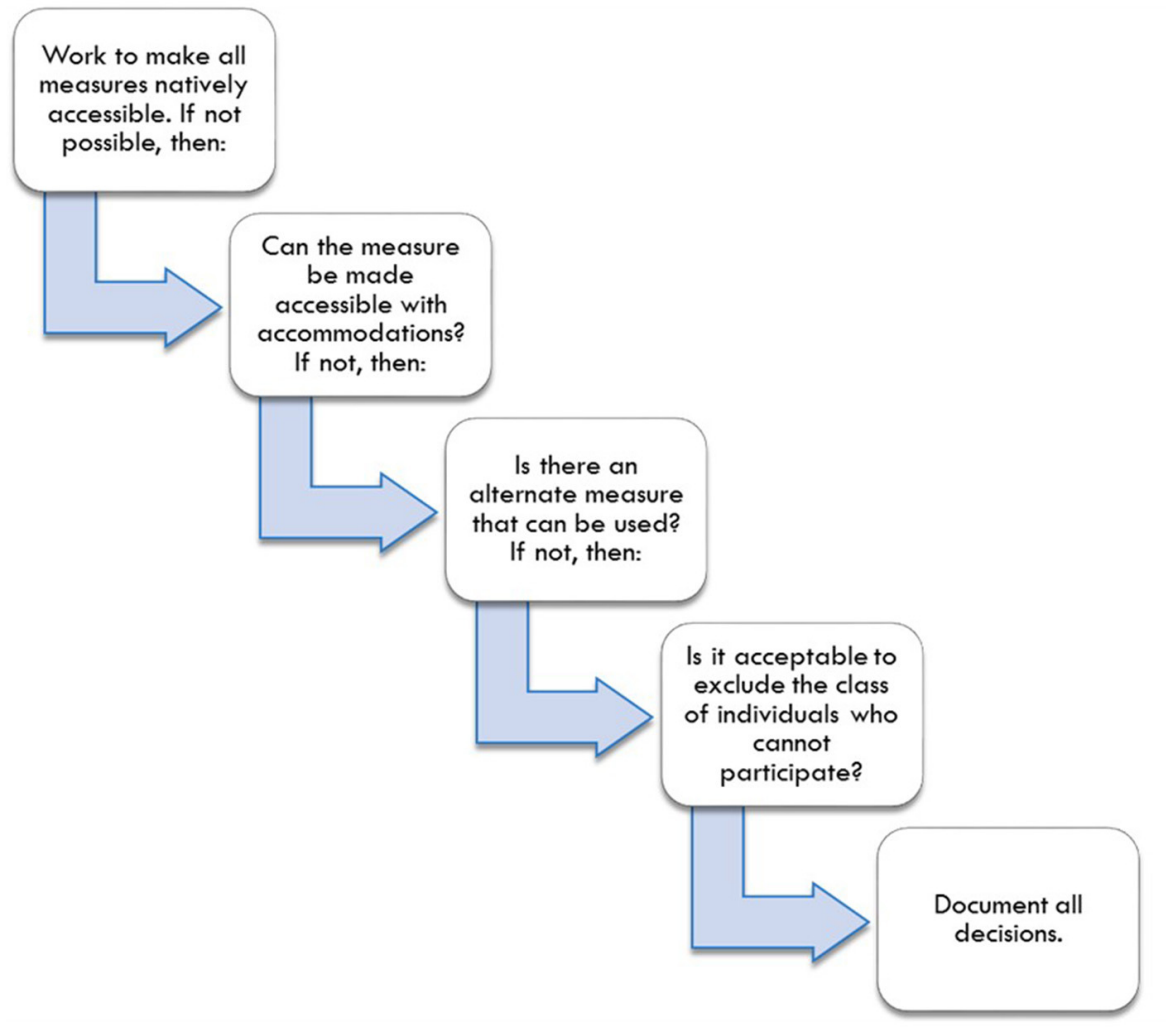
Decision process for creating accessible health research.

The decision process, illustrated in Figure 2, begins with researchers seeking, and test developers designing, measures that are suitable for the broadest range of people and the broadest range of functional limitations found within their population of interest. Second, if measures are not accessible, researchers should plan accommodations and decide whether those accommodations will be coded as standard or non-standard administrations. Researchers should consider what changes to a standardized test administration are acceptable. As AERA (20) notes, standardization is the tradition in testing, but “sometimes flexibility is needed to provide essentially equivalent opportunities for some test takers” (p. 51). In fact, sometimes scores will be more comparable if standardized procedures are changed (e.g., allowing people who are blind to use a Braille version of an assessment). When measures are inaccessible and cannot be made accessible, researchers should plan for the use of alternate measures of the primary construct. Finally, if there are no appropriate alternate measures, researchers should decide, in consensus with relevant stakeholders, whether it is acceptable to exclude the class of individuals who cannot participate.

### The Role of Test Developers

Accommodations and alterations to testing protocols can be expensive when implemented after-the-fact on an individual level to fix a problem with a measure or system. However, when a measure or measurement system is in development, it makes sense to invest in accessibility early in the process to ensure that future users will not have the burden and expense of modifying the assessment tools to meet their needs. We developed an interdisciplinary instrument development process to ensure that accessibility and usability is considered from the start (23). Test developers should follow a process for evaluating accessibility throughout the development process that addresses the following:

1. Integrating accessibility and usability testing as standard components of the instrument development process.
2. Defining the core construct being measured and removing, to the extent possible, secondary task demands that are unnecessary and might reduce accessibility.
3. Using expert review of accessibility as new measures are developed.
4. Prior to finalization of new measures and validation testing, reviewing accessibility through usability testing with people with disabilities who have a wide range of functional abilities to determine whether the measure unfairly disadvantages any group.
5. Once measures are completed, documenting for whom the measure is appropriate (i.e., for whom the measure is accessible) and not.
6. If the measure is not accessible for a sub-group, developing accommodation recommendations or identifying alternate measures.

**Figure 3:**
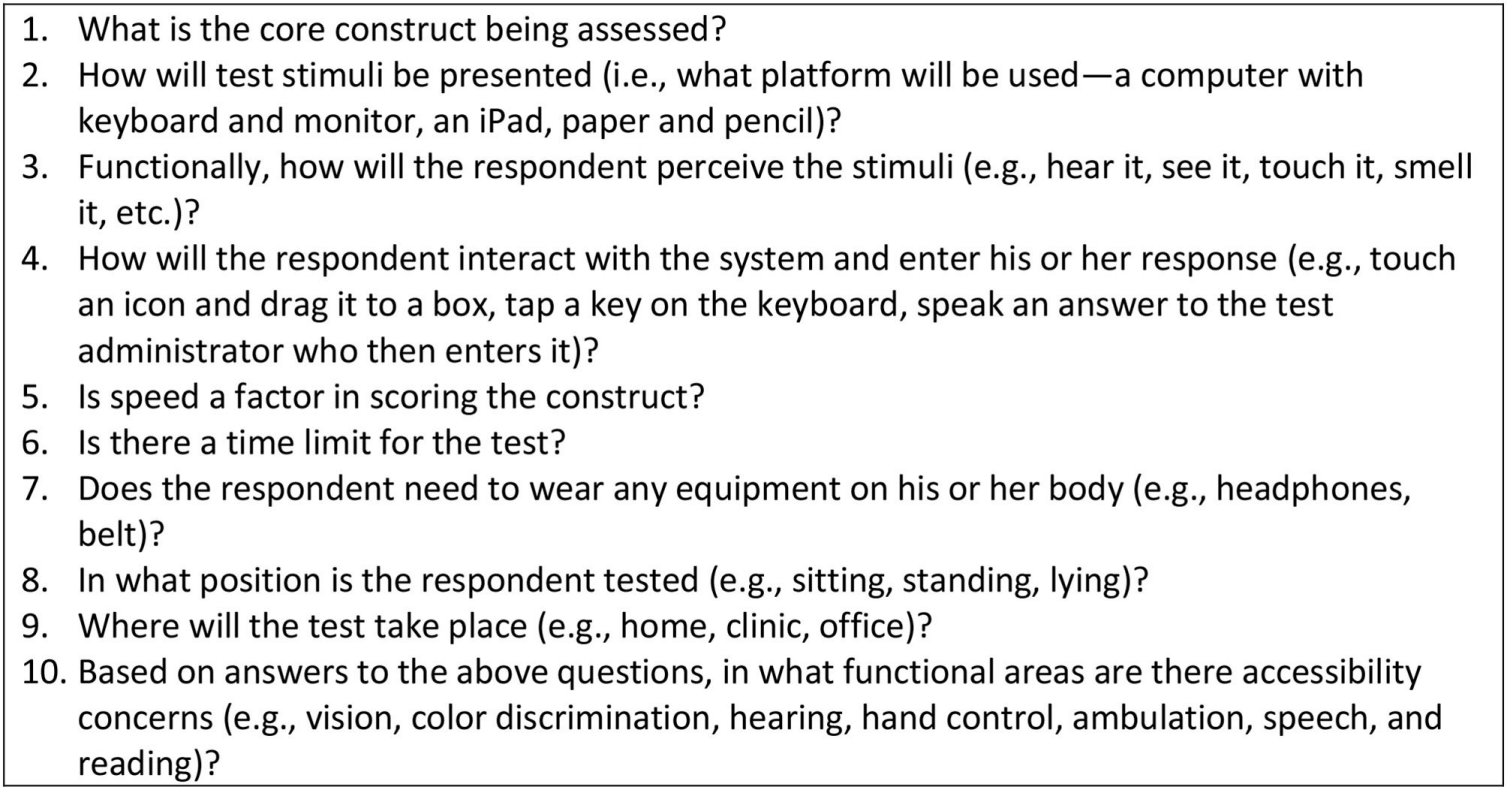
Accessibility questions for test developers.

In Figure 3, we highlight important questions to be answered by test developers as they create accessible measures or measurement systems.

### The Role of Researchers

Researchers interested in including the broadest range of people with disabilities should consider the accessibility of their measures early in the planning process. The primary question for researchers is “How can I ensure my sample remains intact and representative?” The following steps should be considered:

1. Consider the range of functional challenges most likely to occur in a sample and plan ways to ensure inclusion of people with disabilities.
2. Select the most appropriate measures with the broadest accessibility at the beginning of a study.
3. If a measure is not accessible, develop a set of planned accommodations and code them as standard or nonstandard.
4. Build accommodations into a manual so test administrators have clear guidance about what to do when a test cannot be administered as designed.
5. To reduce attrition based on inaccessibility, consider selecting a set of core measures that are accessible to all as part of a larger measurement system. If an individual cannot complete all measures, researchers will still be able to include all participants in the core set of analyses.

Researchers should also collect data to understand better how accessible their measurements systems are by:

1. Tracking the number of participants lost due to inability to conduct measurement.
2. Documenting the rationale for non-standard and failed administrations and analyzing these reasons qualitatively.
3. Considering sub-studies that evaluate the comparability of accommodated vs. non-accommodated test administration, standard vs. non-standard administration, and between primary vs. alternate measures.

**Figure 4:**
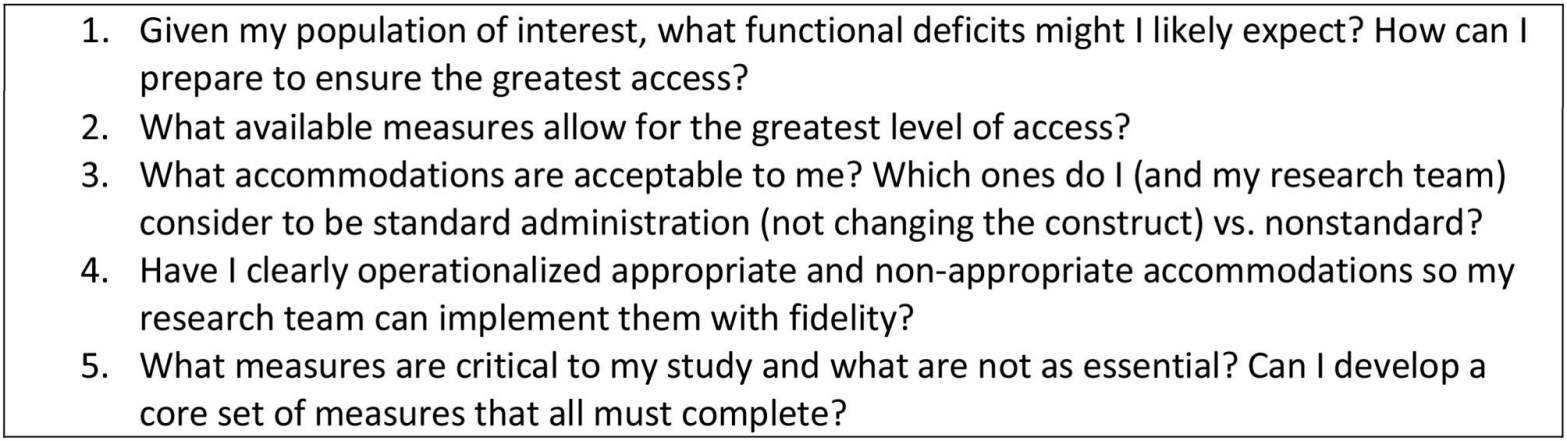
Accessibility questions for researchers.

Figure 4 suggests accessibility issues that researchers should consider.

### The Role of Test Administrators and Research Personnel

Test administrators and other research personnel represent where “the rubber hits the road” in research studies. We have found that their understanding of the purpose of a measure and the purpose of the research affects their ability to implement measurement systems and accommodations appropriately. When facing a person with a disability, test administrators often make informal accommodations that may invalidate the instrument (24). These adjustments are generally not reported to the researchers and become part of measurement error. Clear guidelines are needed to ensure that all test administrators consistently implement the same accommodations.

1. If research personnel do not have experience with people with disabilities, provide training on disability etiquette and opportunities to discuss concerns, fears, or preconceived notions.
2. Screen research participants before they arrive to anticipate the testing challenges they may face. Screening can include informal questions (Here is what we will do, are there any difficulties you might have with these types of tests?) and more formal (e.g., a survey that queries relevant functional abilities). These procedures provide the team with advanced notice about individuals who might need accommodation.
3. If an accommodations manual has been developed, ensure that all research personnel involved in measurement understand accommodations and whether they are considered standard or non-standard administrations. Provide training on how to implement the accommodations with members of the target population (not just mock settings) so that test administrators reach a level of comfort and fluency.
4. Document the accommodations provided and the challenges test administrators encounter in implementing measures (with or without accommodations). Use this information for continuous improvement.

**Figure 5:**
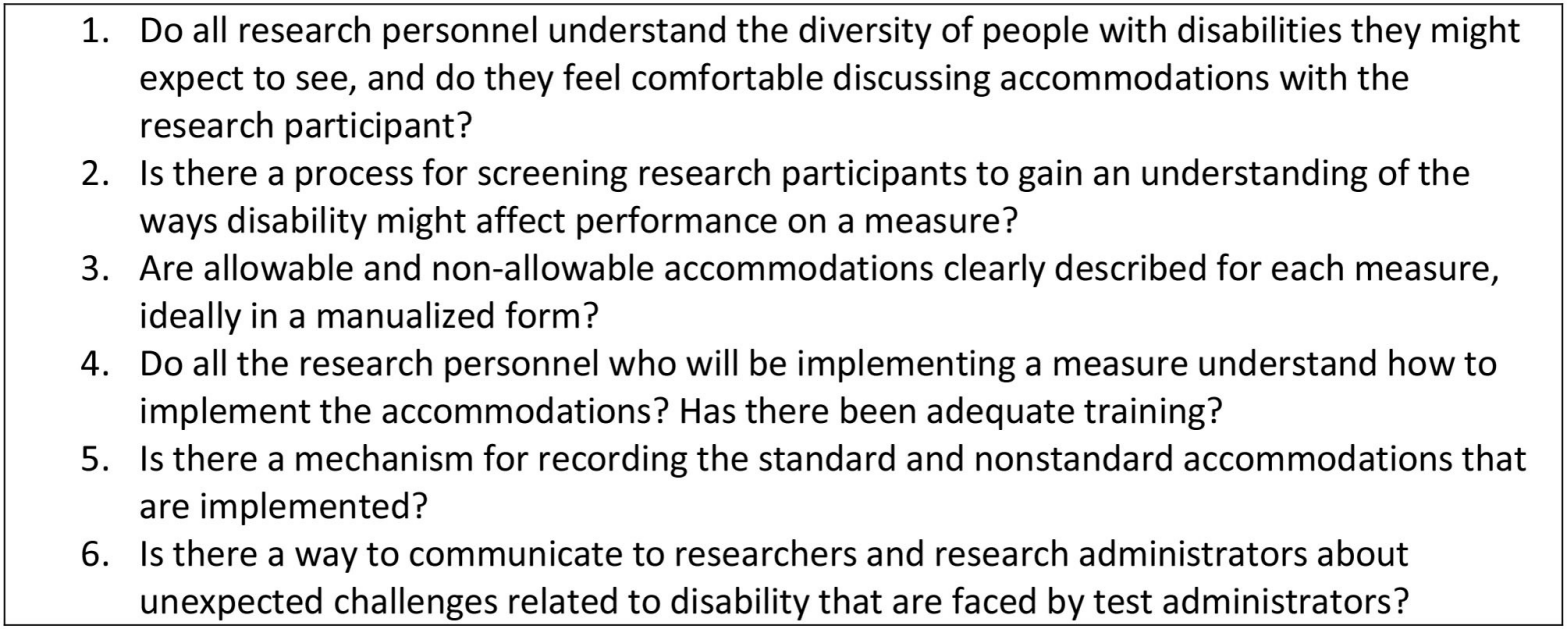
Accessibility questions for test administrators.

Figure 5 suggests accessibility questions that test administrators should ask.

## Conclusion

There is increasing recognition that people with disabilities are vulnerable to inequitable access to health and human services and thus at risk for inferior outcomes (26). People with disabilities must be included in health research and they must be accurately and reliably measured using validated assessment tools (27–29) to reduce these risks. Increasing emphasis on accountability and equity in healthcare, medical rehabilitation, and education underscores the need for valid and reliable measures (29). Comparative effectiveness research, resources and supportive service allocation, and benefits eligibility are predicated on assumptions that scores derived from standardized assessments are valid, sensitive, equivalent, and can be interpreted accurately across diverse populations (30, 31). When people with disabilities are systematically excluded because of inaccessible measurement systems, health research findings are not representative of the population and not useful in decision-making.

## Data Availability Statement

The original contributions presented in the study are included in the article/supplementary material, further inquiries can be directed to the corresponding author/s.

## Author Contributions

MH and SM wrote the article jointly based on work in the NCS as well as previous work on PROMIS and the NIH Toolbox. DS engaged in the NCS work including review of measures, writing White Papers, and helping to conceptualize the decision process. All authors contributed to the article and approved the submitted version.

## Conflict of Interest

The authors declare that the research was conducted in the absence of any commercial or financial relationships that could be construed as a potential conflict of interest.
